# The resting sites and blood-meal sources of *Anopheles minimus *in Taiwan

**DOI:** 10.1186/1475-2875-7-105

**Published:** 2008-06-09

**Authors:** Mei-Chun Chang, Hwa-Jen Teng, Chen-Fu Chen, Yung-Chen Chen, Chian-Ren Jeng

**Affiliations:** 1 Research and Diagnostic Center, Centers for Disease Control, Taiwan; 2 Department of Veterinary Medicine, National Taiwan University, Taipei, Taiwan

## Abstract

**Background:**

The WHO declared Taiwan free from malaria in 1965, but in 2003 the reporting of two introduced cases in a rural area suggested a possible local transmission of this disease. Therefore, understanding the resting sites and the blood sources of *Anopheles minimus *is crucial in order to provide information for implementing vector control strategies.

**Methods:**

During a two-year survey, mosquitoes were collected in houses and their surrounding areas and at the bank of larval habitats by backpack aspirators in 17 villages in rural areas of southern and eastern Taiwan for 1 hr. On the same day, blacklight traps were hung downward overnight. Blood-fed mosquito samples were analysed by PCR.

**Results:**

Of the 195 total households surveyed by backpack aspirators, no *Anopheles *adults were collected inside the houses, while a single *Anopheles minimus *and a single *Anopheles maculatus *were collected outside of the houses. On the same day, 23 *An. minimus*, two *An. maculatus*, two *Anopheles ludlowae*, two *Anopheles sinensis*, and one *Anopheles tessellatus *were collected along the bank of larval habitats. In blacklight traps hung outside of the houses in the villages, 69 *An. minimus*, 62 *An. ludlowae*, 31 *An. sinensis*, and 19 *An. maculatus *were collected. In larval habitats, 98 *An. ludlowae*, 64 *An. minimus*, 49 *An. sinensis*, and 14 *An. maculatus *were collected. Of a total of 10 blood-fed samples, *An. minimus *fed on four animals including bovine (60%), dogs (20%), pig (10%), and non-chicken avian (10%).

**Conclusion:**

*Anopheles minimus*, an opportunist feeder in Taiwan, was not collected inside the houses, but was found outside of the houses in villages and surrounding larval habitats. Therefore, an outdoor transmission of malaria is likely to occur and, thus, the bed nets, which are favoured for controlling the late biting of *An. minimus*, should be a very efficient and effective method for those local residents who sleep outdoors. Additionally, space spray of insecticides for *Anopheles *at night, as well as residual spray inside animal huts and selective larval habitats, are also helpful to control female adults.

## Background

Malaria is documented to have been prevalent throughout much of Taiwan in the19^th ^and 20^th ^centuries. The maximum estimated case number was 1.2 million in 1952 [1]. In November 1946, the International Health Division of the Rockefeller Foundation, in collaboration with the Taiwanese government, established a Malaria Research Center in southern Taiwan in order to instigate a series of antimalaria measures. Among these actions, a four-year island-wide malaria control programme was launched in 1951. The principal control measure was indoor residual house spraying with DDT (0.5–2.0 g of active ingredient per m^2^), which was conducted in conjunction with the larviciding of streams with DDT and automatic flushing of streams. As a result of the success of these actions and patient treatments, the World Health Organization (WHO) declared Taiwan to be free from malaria on December 4, 1965. Furthermore, after 1973, almost all of the reported cases of malaria in Taiwan (22–83 cases per year) were imported [1-3].

Among the 15 Anopheline species that are found in Taiwan, *Anopheles minimus *is regarded as the principal malaria vector [4]. This species (A) is the malaria vector in the Oriental Region and its morphology is similar to its two sibling species, C and E [5,6]. Recently, species C was resolved as *Anopheles harrisoni *by comparing DNA sequence data [7]. Based on DNA analysis of the D3 region of the 28S gene of ribosomal DNA, samples collected from Taiwan are identical to species A [8,9]. Species A has now formally been recognized as *An. minimus *s.s. [10]. More thorough study on molecular identification of *An. minimus *is on going to include samples collected from wider areas. *Anopheles minimus *is able to change its host preference based on host availability and is known as an opportunist feeder [11,12]. The vector status of the secondary species, *Anopheles sinensis*, was questioned by the misidentification of one slide from a 1947–1949 study [4], on which the crithidial flagellates had been misidentified as malaria sporozoites. Of the other 14 Anopheline species in Taiwan, *Anopheles maculatus*, *Anopheles ludlowae*, *Anopheles tessellatus*, *Anopheles jeyporiensis *and *Anopheles annularis *have been implicated in malaria transmission in other countries; however, they are not considered to be potential vectors of malaria in Taiwan [4].

In addition to the imported malaria found in Taiwan, induced malaria also occurred in 1980 (1 case), 1995 (6 cases with 66.67% mortality), and 1997 (1 case) [2]. In August 2003, two cases of locally transmitted malaria (the first since the eradication of malaria in Taiwan) occurred in a rural area of Taitung County. However, only the presence of an imported *Plasmodium *carrier, the existence of competent female vectors (*An. minimus*), and the high-risk behaviour of sleeping outdoors suggest the possibility of mosquito transmission. Since the eradication of malaria in Taiwan, environmental and housing conditions have largely changed. Data on the resting sites and blood-fed hosts of the malaria vectors need to be updated in order to provide information for implementing vector control strategies. The objective of this study is to understand the resting sites and blood-meal sources of *An. minimus *in Taiwan.

## Methods

### Resting site study and mosquito collection

**Figure 1:**
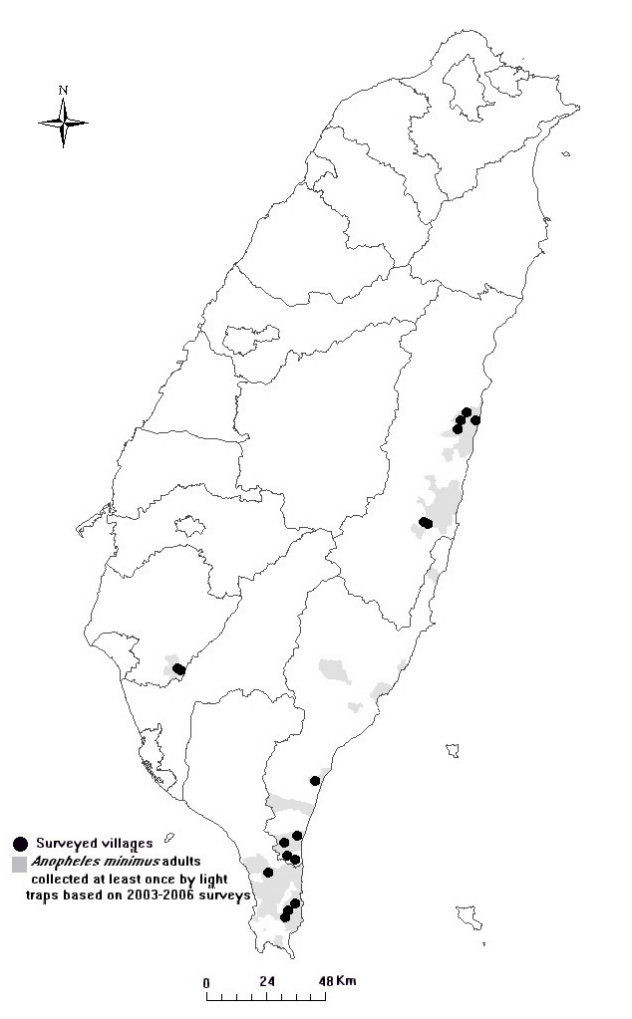
**The survey villages and distribution map of *Anopheles minimus***. Dark solid circles indicate the survey villages in rural areas in southern and eastern Taiwan and the gray areas indicate the collection of *Anopheles minimus *adults (at least once) based on the light trap data from 2003 to 2006 in Taiwan.

The survey villages (in southern and eastern Taiwan) were chosen based on a large number of *An. minimus *adults collected by light traps during the same year or the previous year (Figure 1). Based on the surveys conducted by local health bureaus, the average density (± SD) of *An. minimus *at the study villages comparing with that of all villages examined were 1.80/night/trap (± 8.56) in the study villages and 0.32/night/trap (± 3.18) in all villages examined. From April to September in 2005 and 2006, two to three villages were surveyed each month. On each visit, a larval survey by 14-cm-diameter dippers was conducted along the bank of streams and ditches around or in the surveyed village. A section of a larval habitat was chosen in the morning based on the collection of *An. minimus *larvae or, at least, other *Anopheles *larvae. Two teams collected mosquito adults along the bank and its surroundings for 1 hr during the period between 10:00 and 12:00. Each team included two individuals with one modified CDC backpack aspirator (Model 1412, John W. Hock Company, Gainesville, Florida) and one sweeping net. During the period between 15:00 and 17:00, the same 1 hr collection was also conducted in human dwellings, including inside the houses and their surroundings. The mean number (± SD) of houses sampled per visit was 9.75 (± 1.77). Screen conditions for each surveyed house were recorded. On the same day, one updraft blacklight (UV) trap (Model 1312, John W. Hock Company, Gainesville, Florida) with dry ice was set up downward outside of the houses and the larval habitat, (separately) overnight. All collected mosquitoes were stored in a dry ice box and brought back to the laboratory for species identification. Blood-fed mosquitoes were kept at -20°C for blood meal identification. Additional mosquitoes were collected in animal huts (including pigs, buffalos, and horses) by aspirators or blacklight traps outside of the villages in order to increase the blood-fed mosquito sample.

### Blood-meal identification

Blood-fed mosquitoes were processed individually using the PCR method to identify the blood-meal source. Genomic DNA from blood-fed mosquitoes was extracted using the QIAamp DNA Mini Kit (Qiagen GmbH, Hilden, Germany) and the protocol described by the manufacturer was followed. The same DNA extraction procedure was also applied to blood samples of 10 common animals (i.e., bovine, cat, chicken, dog, goat, horse, human, monkey, pig, and rat) in Taiwan to serve as positive controls and clarify the cross reactions of animal blood. Non-fed mosquitoes served as negative controls. The sensitivity of this test was demonstrated in detection of blood-fed *Aedes aegypti *in a laboratory colony up to five days (1 hr, 1 day, 2 days, 3 days, 4 days, and 5 days) after blood-feeding on a mouse.

The PCR amplifications were conducted in 50 μl of a solution containing 10 mM Tris-HCL (pH 8.3), 50 mM KCL, 1.5 mM MgCl_2_, 2.5 mM dNTP Mix, 1 μM of each primer, 5 units/μl of Tag DNA polymerase (TAKARA BIO Inc., Shiga, Japan), and 1 μl of DNA product. The sequences of the primers for the 10 animals used in the PCR are listed in Table 1. Two additional order-specific primers (mammalian and avian) were included to detect other possible hosts as well. Reactions began with an incubation at 94°C for 2 min, followed by 35 cycles consisting of 94°C at 30 sec, 54–70°C at 30 sec (detailed temperature for each primer listed in Table 1), and 72°C at 30 sec. The reaction was completed by incubation at 72°C for 20 min and kept at 4°C. Then, 17 μl samples of PCR products were analyzed using a 2% agarose gel in Tris Borate EDTA and visualized on a UV light box after ethidium bromide staining. Negative and positive controls were included in each PCR.

**Table 1:** Order-specific group primers and species-specific primers used in blood-meal identification

Test animal	Primer sequence^1 ^(F: forward 5'-3' and R: reverse 5'-3')	Annealing temp (°C)	Size of amplified products (bp)
Avian	F: GACTGTGACAAAATCCCNTTCCA R: GGTCTTCATCTYHGGYTTACAAGAC	64	508
Mammalian	F: CGAAGCTTGATATGAAAAACCATCGTTG R: TGTAGTTRTCWGGGTCHCCTA	59	772
Chicken	F: GGGACACCCTCCCCCTTAATGACA R: GGAGGGCTGGAAGAAGGAGTG	69	266
Bovine	F: GCCATATACTCTCCTTGGTGACA R: GTAGGCTTGGGAATAGTACGA	61	271
Pig	F: GCCTAAATCTCCCCTCAATGGTA R: ATGAAAGAGGCAAATAGATTTTCG	64	212
Goat	F: TTAAAGACTGAGAGCATGATA R: ATGAAAGAGGCAAATAGATTTTCG	54	225
Cat	F: TTCTCAGGATATACCCTTGACA R: GAAAGAGCCCATTGAGGAAATC	60	180
Dog	F: GAACTAGGTCAGCCCGGTACTT R: CGGAGCACCAATTATTAACGGC	67	153
Horse	F: CCCTAAGCCTCCTAATCCGT R: AGGAATGATGGGGGAAGTAA	56	235
Human	F: TTCGGCGCATGAGCTGGAGTCC R: TATGCGGGGAAACGCCATATCG	70	228
Monkey	F: CCTCTTTCCTGCTGCTAATG R: TTTGATACTGGGATATGGCG	62	222
Rat	F: CGGCCACCCAGAAGTGTACATC R: GGCTCGGGTGTCTACATCTAGG	67	196

^1^The primer sequences were cited from papers of Ngo and Kramer [22], Lahiff *et al *[23] and Parodi *et al *[24].

### Statistical analysis

Data were transformed by square root of (x+0.5) prior to analysis to meet the assumptions of t-test [13]. Because of the dependence of the data at the same collection site, paired *t *tests were used to compare differences among the number of *Anopheles *mosquitoes collected at different collection sites under different collection methods. Furthermore, the number of *Anopheles *mosquitoes collected against the number of *Armigeres*, *Aedes*, and *Culex *under different collection sites and methods were also compared by this test.

## Results

### Resting site study

**Figure 2:**
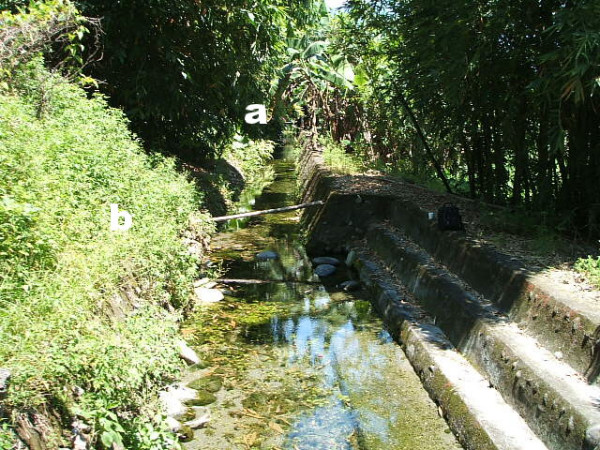
**Typical resting sites of *Anopheles minimus *adults in a ditch in Hwalien County, Taiwan**. a: the collection site of *Anopheles minimus *(5 females) in 2005 with a native fern (*Asplenium antiquum *Makino) and b: the collection site of *An. minimus *(12 females and five males) in 2006 with a native plant (*Bidens pilosa *L.).

After a two-year survey in 17 villages (20 visits), with a total of 195 households surveyed, significantly more *Anopheles *mosquitoes were collected by blacklight traps than by backpack aspirators in human dwellings (*t*_19 _= 3.59, *P *< 0.01) but not in larval habitats (*t*_19 _= 2.00, *P *> 0.05). No differences were found in locations by the same collecting methods (*t*_19 _= 0.11 and 1.61, *P *> 0.05). No *Anopheles *adults were collected inside the houses while a single *An. minimus *and a single *An. maculatus *were collected outside of the houses (Table 2). At the same time, 23 *An. minimus*, two *An. maculatus*, two *An. ludlowae*, two *An. sinensis*, and one *An. tessellatus *were collected along the bank of larval habitats. Most of the *Anopheles *adults (27 out of 30) were collected in one location over two years (Figure 2). In 2005, *An. minimus *(five females), *An. tessellatus *(one female), *An. sinensis *(two males), *Culex quinquefasciatus *(six females and two males), *Culex tritaeniorhynchus *(five females and four males), *Culex annulus *(two females), *Culex fuscocephala *(one female), *Armigeres subalbatus *(one female and two males), and *Aedes albopictus *(one male) were collected at the same time and the dominant plant was a native fern (*Asplenium antiquum*). In 2006, *An. minimus *(12 females and five males), *An. ludlowae *(two females), *Cx. annulus *(12 females and one male), *Cx. tritaeniorhynchus *(seven females), and *Ae. albopictus *(three females) were collected and the dominant plant was a native *Bidens pilosa*. In light traps hung outside of the houses in human dwellings, 69 *An. minimus*, 62 *An. ludlowae*, 31 *An. sinensis*, and 19 *An. maculatus *were collected, while 98 *An. ludlowae*, 64 *An. minimus*, 49 *An. sinensis*, and 14 *An. maculatus *were collected in larval habitats.

**Table 2:** Number of mosquitoes collected using different collection methods in 20 visits (17 villages of 4 counties) Taiwan, 2005–2006

	CDC backpack aspirators^1^				Blacklight traps^1^		
		
Mosquito species	Human dwelling			Larval habitat	Human dwelling (outdoor)	Larval habitat	Total
					
	Indoor^2^	Outdoor	Total				
*Anopheles *species	0	2	2	30	181	225	438
*An. minimus*	0	1	1	23	69	64	157
*An. maculatus*	0	1	1	2	19	14	36
*An. ludlowae*	0	0	0	2	62	98	162
*An. sinensis*	0	0	0	2	31	49	82
*An. tessellatus*	0	0	0	1	0	0	1
*Culex *species	73	135	208	66	386	187	847
*Aedes *and *Ochlerotatus *species	14	115	129	113	260	1,675	2,177
*Armigeres *species	87	229	316	5	187	5	513
Other species	0	0	0	1	8	2	11
Total	174	481	655	215	1,022	2,094	3,986

^1^In each visit, two backpack aspirators were used to collect inside and outside of the houses for 1 hr and a section of a larval habitat for 1 hr, respectively. On the same day, one blacklight trap hung downward overnight to collect adults outside of the houses and the bank of the larval habitats, respectively. ^2^The house number surveyed was 195.

Significantly more *Armigeres *(*t*_19 _= 3.09, *P *< 0.01), *Aedes *(*t*_19 _= 2.88, *P *< 0.01) and *Culex *(*t*_19 _= 2.37, *P *< 0.05) adults were collected indoors by backpack aspirators than *Anopheles *mosquitoes. Similar results were found for backpack aspirators used outdoors (*Armigeres *vs. *Anopheles t*_19 _= 4.78, *P *< 0.001;*Aedes *vs. *Anopheles t*_19 _= 5.70, *P *< 0.001; *Culex *vs. *Anopheles t*_19 _= 3.06, *P *< 0.01). The dominant mosquito species collected outdoors in human dwellings by backpack aspirators were *Ar. subalbatus *(47.6%), *Cx. quinquefasciatus *(26.4%), and *Ae. albopictus *(23.7%). Five other mosquito species found in small numbers were *Cx. tritaeniorhynchus *(1.2%), *Cx. annulus *(0.4%), *An. minimus *(0.2%), *An. maculatus *(0.2%), and *Aedes vexans vexans *(0.2%). Of the 174 mosquitoes collected indoors, only *Ar. subalbatus *(50%), *Cx. quinquefasciatus *(42%), and *Ae. albopictus *(8%) were present. No difference of *Aedes*, *Armigeres*, and *Culex *against *Anopheles *adults (*t*_19 _= 0.03–1.85, *P *> 0.05) was found for data in larval habitats collected by backpack aspirators or blacklight traps or in human dwellings collected by blacklight traps.

In addition, the screen conditions of each house surveyed in 2006 (a total of 85 houses) were checked. Half (52%) of surveyed houses had completely screened doors and windows, 15% of the surveyed houses were partially screened, and 33% of the surveyed houses were not screened (Table 3).

**Table 3:** Window and door screens in the households of rural areas, Taiwan, 2006

			Screens on window and door					
			
Location	Village no.	House no.	Complete		Partial^1^		No	
			
			House	%	House	%	House	%
Hwa-lien	4	36	20	56%	6	17%	10	28%
Tai-tung	2	24	12	50%	3	13%	9	38%
Ping-tung	2	17	6	35%	4	24%	7	41%
Tainan	1	8	6	75%	0	0%	2	25%
Total	9	85	44	52%	13	15%	28	33%

^1^Partial indicate that at least one window or door was not closed in the survey (i.e., in the afternoon 15:00–17:00).

### Blood-meal identification

**Figure 3:**
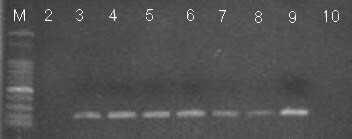
**Analysis of bloodmeal on blood-fed *Aedes aegypti *females in a laboratory colony up to five days after blood-feeding on a mouse by PCR (196 bp product)**. Lane M, DNA molecular weight marker; lane 2 before fed; lane 3 1 hr after blood-fed; lane 4 1 day after blood-fed; lane 5 2 days after blood-fed; lane 6 3 days after blood-fed; lane 7 4 days after blood-fed; lane 8 5 days after blood-fed; lane 9 mouse blood; lane 10 negative control.

Figure 3 showed the results for the sensitivity of the PCR test in detection of blood-fed *Ae. aegypti *in a laboratory colony up to five days (Lane 3 to 8) after blood-feeding on a mouse. The expected 196 bp PCR product was detected in all the samples collected 1 hr, 1 day, 2 days, 3 days, 4 days, and 5 days after blood-feeding. Among 10 blood-fed *An. minimus*, six females (60%) fed on bovine, two females (20%) on dog, one female (10%) on pig, and one female (10%) for non-chicken avian (Table 4). Among 44 blood-fed *An. sinensis*, almost 86.4% of the females (38 females) fed on pig, followed by bovine (9.1%) and horse (4.5%). Three and one *An. maculatus *females fed on bovine and dog, respectively. *Anopheles ludlowae *fed on bovine (two females) and horse (one female). A single *An. tessellatus *female fed on bovine. Human, dog, pig, bovine, horses, and non-chicken avian were detected in *Culex *or *Aedes *blood-fed samples.

**Table 4:** Sources of origin for mosquito blood-meals determined by PCR assays

Mosquito species	Total	Human	Dog	Pig	Bovine	Horse	Non-chicken avian
*Anopheles minimus*	10	0	2	1	6	0	1
*Anopheles sinensis*	44	0	0	38	4	2	0
*Anopheles maculatus*	4	0	1	0	3	0	0
*Anopheles ludlowae*	3	0	0	0	2	1	0
*Anopheles tessellatus*	1	0	0	0	1	0	0
*Culex tritaeniorhynchus*	27	1	1	21	1	1	2
*Culex annulus*	3	1	1	0	1	0	0
*Culex fuscocephala*	1	0	0	0	1	0	0
*Culex bitaeniorhynchus*	1	1	0	0	0	0	0
*Aedes albopictus*	5	2	0	3	0	0	0
*Culex quinquefasciatus*	2	0	0	0	0	2	0
Total	101	5	5	63	19	6	3

## Discussion

In this study, no *Anopheles *adults were collected inside the houses but two *Anopheles *adults and a large number of *Anopheles *(including *An. minimus*) were collected outside of the houses by backpack aspirators and blacklight traps, respectively. The principal malaria vector, *An. minimus*, fed on four animals, including bovine, dog, pig, and non-chicken avian. Therefore, an outdoor transmission of malaria is likely to occur.

No *Anopheles *adults were collected inside the houses while the surveyed houses were not fully screened (15% partial screens plus 33% no screens). Significantly more *Ar. subalbatus *and *Ae. albopictus *were collected inside the houses. These two species are dusk or day biters, making local residents close the door and windows at sunset or use repellents/insecticides to prevent mosquito bites. These behaviours of local residents gave late night species no chance to enter the houses and bite people. The biting rhythm of *An. minimus *was different by location as a result of the local condition. In Lang Nhot, Central Vietnam [12] and Taiwan [14], this species was a late night biter. Hence, in Taiwan, the behaviour of local residents prevented these mosquitoes from entering the house. Furthermore, this species will not rest on indoor surfaces after biting.

The indoor and outdoor biting activities of *An. minimus *are varied by location and surveyed time [11,15]. The surveys in Rattanakiry, Cambodia and Vientiane, Lao in 1999 showed that the biting density at outdoor collection was higher than indoor in hot season (March) while the opposite result was found in cool season (October) [15]. In this study, the survey was conducted only in one hot season (April to September). In order to conclude the low possibility of indoor malaria transmission in Taiwan, the same study in cool season should be conducted to clarify this point. Additionally, the sampling strategy of this study biased on outside collections, which only one 1 hr-indoor collection in late afternoon was made. This indoor collection in daytime referred to the resting population only. No information on the indoor biting activity of these mosquitoes at night was available in this study. There is a possibility that *An. minimus *females enter the house, blood feed on human, and exit from the house to the bank of the nearby breeding sites within one night. Further study should be conducted to clarify this point before any solid conclusion can be made on low possibility of indoor malaria transmission in Taiwan.

In this study, *An. minimus *fed on four animal's blood. Therefore, these results suggest that the local population of *An. minimus *is an opportunist feeder in Taiwan. All blood fed samples of *An. minimus *except one female (collected from a pig farm) were collected by backpack aspirators or blacklight traps. However, the host-feeding pattern described refers only to the relative frequency of blood source detection in the blood-meal samples, which does not necessarily imply a higher preference for a particular host. In Southeast Asia, *An. minimus *was either zoophilic or anthropophilic, depending on the local host availability [11,12]. The host preferences of *An. minimus *in Taiwan reported in 1933 [16] were bovine (68.5%), human (25.2%), chicken (3.5%), and pig (2.8%). In comparing the current results with those of 1933, both studies detected bovine and pig. No human and chicken but, instead, dog and non-chicken avian were detected. Because dogs live very close to their owners and *An. minimus *females were found outside of the houses in human dwellings, outdoor transmission is still possible, such as in the cases that occurred in 2003. The patients slept outdoors overnight.

Three malaria control measures directed towards adult mosquitoes were considered in malaria epidemic areas [17]. The first was indoor residual spraying, a treatment that can effectively control epidemics but only when implemented at an early stage of the outbreak and not after the epidemic's peak [18]. In this study, *Anopheles *adults were only collected outside of the houses and surrounding larval habitats. Furthermore, this species fed on the blood of four animals. Therefore, an indoor residual spraying in houses would not be effective; instead, this measure should be applied to animal huts and surrounding larval habitats. However, in this study, only one surrounding larval habitat was suitable for spraying; therefore, the application sites of larval habitats should be carefully evaluated. The second measure was space spraying. Because more *Anopheles *adults were collected in light traps in the results, a space spray at night would be a good control measure to kill host-searching females. The last was the use of insecticide-treated bed nets, which had been shown to significantly reduce malaria morbidity and mortality in malaria epidemic areas [19-21]. Additionally, a good surveillance and the proper management of malaria imported cases and patient movement is also important to prevent the reintroduction of malaria into Taiwan. Furthermore, routine vector surveillance will provide the valuable information on the trends in vector dynamics and behaviour.

## Conclusion

*Anopheles minimus*, an opportunist feeder in Taiwan, was not collected inside the houses but were found outside of the houses by backpack aspirators and light traps. Therefore, an outdoor transmission of malaria is likely to occur and, thus, the bed nets, which are favoured for controlling the late biting of *An. minimus*, should be a very efficient and effective method for those local residents who sleep outdoors. The use of space spray of insecticides for *Anopheles *at night, as well as residual spray in animal huts and selective larval habitats, are helpful to control adults. Additionally, a good surveillance and the proper management of malaria imported cases and patient movement is also important to prevent the reintroduction of malaria into Taiwan.

## Authors' contributions

MCC carried out the blood-meal identification, collected field data, and helped draft the manuscript, HJT designed the survey, collected field data, performed the statistical analysis, drafted, and polished the manuscript, CFC and YCC provided significant input on data collection in the field and helped draft the manuscript, CRJ participated in the design of the study and critically revised the manuscript. All authors read and approved the final manuscript.
